# Observation of a Polar Compound with Halide Ordering in the Compositional Series (CH_3_NH_3_)_2_Te(Br_*x*_Cl_1–*x*_)_6_

**DOI:** 10.1021/acs.inorgchem.5c03380

**Published:** 2025-10-22

**Authors:** Yuhan Liu, Prajna Bhatt, Roxy Lee, Avishek Dey, Anna Regoutz, Robin S. Perry, Robert G. Palgrave

**Affiliations:** † Department of Chemistry, 4919 University College London, 20 Gordon St, London WC1H 0AJ, U.K.; ‡ Department of Chemical Engineering, University College London, Torrington Place, London WC1E 7JE, U.K.; § Istituto Officina dei Materiali (IOM)-CNR, Laboratorio TASC, In Area Science Park, S.S.14, Km 163.5, Trieste I-34149, Italy; ∥ Department of Energy Conversion and Storage, Danmarks Tekniske Universitet, Fysikvej, 310, 334 2800 Kgs. Lyngby, Denmark; ⊥ London Centre for Nanotechnology, University College London, London WC1H 0AH, U.K.; # Department of Chemistry, 6396 University of Oxford, Inorganic Chemistry Laboratory, South Parks Road, Oxford OX1 3QR, U.K.; ∇ Department of Physics and Astronomy, University College London, London WC1E 6BT, U.K.

## Abstract

Single-crystal samples of compounds in the series (CH_3_NH_3_)_2_Te­(Cl_1–*x*_Br_*x*_)_6_ were produced with counter-diffusion crystal growth in silica gels, and new crystal structures are determined using single-crystal X-ray diffraction. With increasing Br content, the structure transitions from *P*3̅*m*1 to *P*6_3_ *mc* to *R*3̅*m* and finally to *Fm*3̅*m*. The compound with x≈0.068 adopts the polar *P*6_3_ *mc* space group, and the structure has partially ordered Cl^–^ and Br^–^ ions. The crystals of this compound show distinct piezoelectric domains with different responses by Piezoresponse Force Microscopy (PFM), confirming their polar structure.

## Graphical abstract

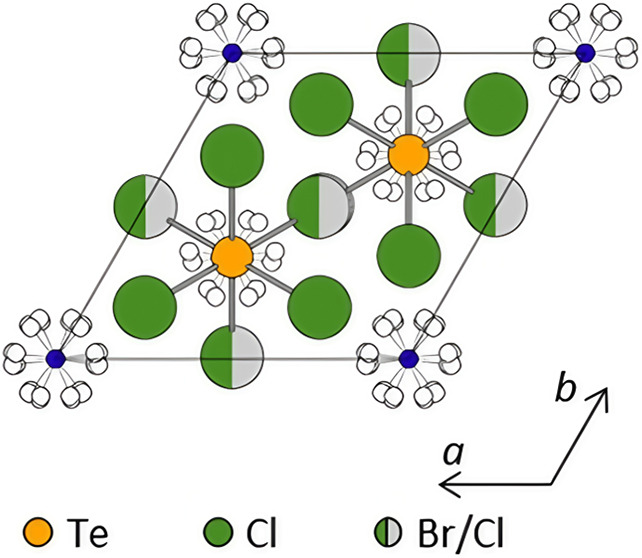

## Introduction

Complex halide materials, containing halide anions and at least two different cations, as exemplified by halide perovskites, have proven to be extremely interesting and valuable materials undergoing renewed interest in recent years. Polar complex halides, like their well-studied oxide counterparts, may exhibit additional important properties such as piezoelectricity, ferroelectricity, effective charge separation, and high ionic conductivity, driven by their noncentrosymmetric crystal structures.^1^ The synthesis of polar halide compounds with these properties may impact the current uses of complex halides in photovoltaics and optoelectronic materials or may enable new functionalities. Although the vast majority of halide perovskite and related compounds adopt centrosymmetric structures, some strategies have emerged to engineer noncentrosymmetric complex halide compounds.

The use of polar molecular cations at the A-site is a versatile method to introduce polar properties into complex halides.^2^ Chiral halide perovskites can be produced using chiral molecular ions, which have been extensively studied.^3^ At low temperatures, CsSnBr_3_ has been found to crystallize in a polar space group driven by the asymmetry of the Sn­(II) lone-pair cation.^4,5^ CsGeBr_3_ is also polar for similar reasons, and ACuCl_4_ compounds, where A is a chlorinated organic molecular ion, are polar due to the orientation of the molecular ions.^6,7^

Anion ordering is another promising route for the formation of polar structures. Charles et al. studied anion ordering in oxy-fluoride double perovskites (A_2_BMO_*x*_F_6–*x*_).^8^ There it was found that structural distortions, such as octahedral rotations, could couple with anion order to produce a large variety of nonpolar, polar, and chiral-polar structures. Anion order among halide (rather than oxy-halide) materials appears to be rare. Mixed bromide–iodide two–dimensional (2D) lead Ruddlesden–Popper phases were shown to display anion ordering, with Br appearing in the apical sites and I in the bridging sites of the 2D Pb halide layer.^9^ In the triple perovskite A_3_B_2_X_9_ series, bromide and iodide ordering has again been observed, which is driven by differences in the bridging and apical anion positions.^10^ Due to the symmetrical nature of the Br–I ordering in both these cases, neither structure was found to be polar.

Another important complex halide family of compounds is the vacancy-ordered double perovskites with chemical formula A_2_BX_6_.^11–14^ The A_2_BX_6_ family is characterized by isolated [BX_6_]^2–^ octahedra and is very commonly found in the *Fm*3̅*m* aristotype structure.^15^ Lower symmetry structures result from distortions of the aristotype through correlative rotations of the [BX_6_]^2–^ octahedra and distortion of the cubic lattice, but some other A_2_BX_6_ structures are not subgroups of the cubic parent.^16^ Hybrid A_2_BX_6_ compounds are those with molecular ion A-site cations, and add another layer of structural complexity.^17–20^ The crystal structures of different hybrid A_2_BX_6_ compounds with organic cations were previously reported, and many of them undergo a structural phase transition at various temperatures.^21–23^

In a previous work, we reported the phase transitions of MA_2_TeCl_6_ (MA = CH_3_NH_3_^+^) at variable temperatures.^22^ Here, we study the crystal structures of the MA_2_Te­(Cl_1–*x*_Br_*x*_)_6_ series at room temperature. We demonstrate that careful control of the anion ratio leads to the formation of a polar structure containing partially ordered halide anions that is stable only in a narrow compositional range. We believe that this is the first report of a polar compound in which the polarity exists by virtue of halide ordering. This observation illustrates how the delicate balance between the ion sizes in A_2_BX_6_ compounds can lead to diverse physical properties.

## Experimental Section

### Single-Crystal Growth

The synthesis of MA_2_Te­(Cl_1–*x*_Br_*x*_)_6_ was first attempted using solution-based coprecipitation and solid-state synthesis in a sealed ampule. However, neither approach resulted in a pure phase for low-Br MA_2_Te­(Cl_1–*x*_Br_*x*_)_6_ (see SI).

Instead, MA_2_Te­(Cl_1–*x*_Br_*x*_)_6_ single crystals were synthesized using the counter-diffusion crystal growth (CDCG) method in a silica hydrogel. TeCl_4_ solution was first prepared by adding TeO_2_ (Sigma-Aldrich, 99%) powder to 37 wt % HCl (Sigma-Aldrich). The solution was stirred for 1 h at 100 °C. Then, TeBr_4_ powder (Alfa Aesar, 99.9%) was added to the cooled TeCl_4_ solution based on the required stoichiometry to obtain a TeX_4_ (X = Br, Cl) solution with the desired Br/Cl ratio. Finally, 2 mL of a 0.6 M aqueous solution of Na_2_SiO_3_ (Sigma-Aldrich) was added dropwise to the TeX_4_ solution (∼10 mL) under stirring. The beaker was then covered with parafilm and placed in a low-temperature oven at 29 °C for 48 h to form a TeX_4_ silica gel.^24,25^

Methylamine solution (Sigma-Aldrich, 40 wt % in H_2_O) was reacted with 37 wt % HCl (Sigma-Aldrich) at room temperature, and the resulting solution was stirred at 110 °C to precipitate MACl powder. After filtering and washing with diethyl ether (Fisher Chemical, 99.5%), the MACl precipitate was dried at 60 °C to produce a white powder. The MACl powder was then redissolved in DI water to form a saturated solution, which was carefully layered atop the previously prepared TeX_4_ containing silica gel surface. Crystal formation was be observed through the gel, occurring between 4 and 7 days. All crystals were picked out using tweezers and dried overnight on filter paper in air. The CDCG method was conducted multiple times to ensure the reproducibility of the synthesis method with nominal 7 and 13% Br for the low-Br and high-Br compounds, respectively. The rationale for these concentrations are discussed in the Supporting Information.

### DFT Calculations

Density functional theory (DFT) calculations were performed within periodic boundary conditions via the Vienna *Ab initio* Simulation Package (VASP).^26^ The projector-augmented wave (PAW) method was used to describe the interaction between the core and valence electrons.^27,28^ A version of the Perdew–Burke–Ernzehof (PBE)^29^ functional that is adapted toward the description of solid state systems, PBEsol,^30^ was used in this work. A Γ-centered 3 × 3 x 3 *k*-point mesh and a plane-wave cutoff energy of 350 eV were found to converge the total energy of all structures to within 1 meV per atom. The plane-wave cutoff energy was increased to 455 eV for geometry optimization calculations to avoid the possibility of Pulay stress.^31^

The Raman spectra were simulated using the methodology of Porezag et al.^32^ To calculate the off-resonance Raman activity of a mode, the derivative of the polarizability with respect to the normal mode coordinate was computed using phonons at the Γ-point and macroscopic dielectric tensor from density functional perturbation theory (DFPT). The structures were relaxed with a tight ionic force criterion of 1 × 10^–5^ eV A^–1^ to increase the accuracy when calculating atomic forces and phonon frequencies. For the DFPT calculations, convergence with respect to the *k*-point density and plane-wave energy cutoff was confirmed for the ionic contribution to the static dielectric constant, ∈_ionic_. The off-resonance Raman activities were evaluated using the vasp_raman.py code.^33^ To simulate the experimental line shape, Gaussian (2.0 eV) and Lorentzian (4.0 eV) broadening were applied to the simulated Raman spectra using the Galore software package.^34^ The choice of alloy structures for calculated Raman spectra was previously determined by Karim et al. and is applied here as well.^35^

### Characterization

Powder X-ray diffraction (PXD) was performed using a Stoe Stadi-P X-ray diffractometer with a Cu Kα_1_ radiation source (λ = 1.5406 Å, 40 kV, 30 mA) in transmission mode. Data were collected from 5 to 50° at 0.5° per step for 15 s.

Single-crystal X-ray diffraction (SXD) was performed on an Agilent SuperNova diffractometer at 295 K. Data were collected with a Mo Kα X-ray source (λ = 0.7107 Å) and processed with CrysAlisPro,^36^ full spheres of data were collected to 0.8 Å resolution using 1° scan frames in ω. The crystal structure was solved using SHELXT and refined with SHELXL within the Olex2 software suite.^37–39^ The refinement procedure forthe MA^+^ cation was adapted from previous work.^22^ Average bond angles, bond lengths, and projection distances between the halide and Te layers were calculated using Mercury^40^ from the atomic coordinates derived from the SXD results. Bond angle variance and pseudocubic lattice parameters are calculated by VESTA.^41^

Raman spectra were obtained using a Renishaw 633 nm Raman spectrometer with a 50× objective lens. The spectral resolution was 4 cm^–1^ with a step size of 0.5 cm^–1^.

X-ray photoelectron spectroscopy (XPS) was performed using a Thermo Scientific Kα spectrometer, which uses a monochromatized Al K_α_ X-ray source (*hν* = 1486.6 eV), a hemispherical analyzer, and a two-dimensional detector. The electron energy analyzer consists of a double focusing 180° hemisphere with a mean radius of 125 mm, operated in constant analyzer energy (CAE) mode, and a 128-channel position sensitive detector. Measurements were conducted with a 400 μm spot size using a dual-beam flood gun (electron and Ar^+^ ion) with a 100 mA current. The pass energy was 200 eV for the survey spectra and 40 eV for the core levels. The Thermo Avantage v5.9925 software package was used for XPS spectral analysis, and all spectral fittings were performed with a Shirley background.

Piezoresponse force microscopy (PFM) was carried out using a Bruker Dimension Icon atomic force microscope. To detect any piezoresponse in the crystals, an AC voltage with an amplitude of 3 V peak-to-peak near the contact resonance frequency was applied to the samples. The crystals were stuck to the AFM stage using conductive carbon tape. Bruker SCM-PIT-V2 silicon AFM probes with a Pt/Ir conductive coating and a nominal stiffness of 3 N m^–1^ were used for the measurements.

## Results and Discussion

Four distinct room-temperature structures were found in the series MA_2_Te­(Cl_1–*x*_Br_*x*_)_6_ (0 ≤ *x* ≤ 1), including the known structures of the end members (*x* = 0,1), as shown in Figure S1. The PXD patterns of crushed CDCG single crystals are shown in Figure 1, and clearly indicate the different crystal structures of the two mixed-halide MA_2_Te­(Cl_1–*x*_Br_*x*_)_6_ samples (labeled as low-Br and high-Br), in comparison to MA_2_TeCl_6_ (trigonal, *P*3̅*m*1) or MA_2_TeBr_6_ (cubic, *Fm*3̅*m*).^22,42^ X-ray photoelectron spectroscopy (XPS) shows that the low-Br sample contains about 6.8% Br, while the high-Br sample contains 10.1% Br (see Table S2 and Figure S1). A similar composition-driven structural transition for organic hexaiodoplatinates, A_2_PtI_6_, has been previously reported.^43^ SXD was then performed to examine the crystal structures of the two new phases.

**Figure 1:**
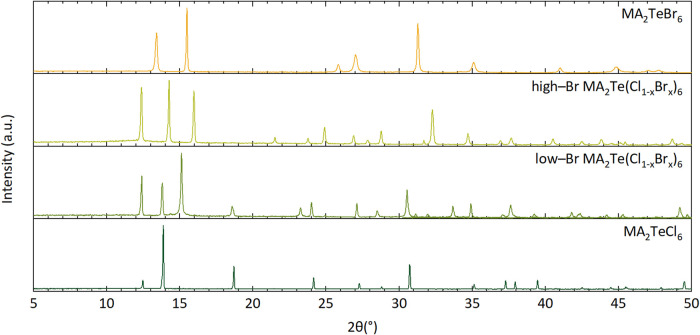
PXD patterns of the room-temperature MA_2_Te­(Cl_1–*x*_Br_*x*_)_6_ phases (λ = 1.5406 Å).

### Crystal Structures of MA_2_TeCl_6_ and MA_2_TeBr_6_

The structures of MA_2_TeCl_6_ and MA_2_TeBr_6_ have been previously reported, and we summarize the main features here to aid in the discussion of the new compounds.^22,44^

MA_2_TeBr_6_ crystallizes in the *Fm*3̅*m* space group, with lattice parameter *a* = 11.445(8) Å, a regular octahedral environment around Te, and a uniform Te–Br bond distance of 2.712(2) Å. The cell volume per formula unit is 374.8 Å^3^. We consider this the aristotype structure from which most of the other structures discussed below can be derived by the distortion and rotation of the octahedra.

MA_2_TeCl_6_ crystallizes in the *P*3̅*m*1 space group, a subgroup of *Fm*3̅*m*, which can be reached by a rotation of the [TeCl_6_]^2–^ octahedra around each cubic axis, in this case by around 5.3°, and a slight distortion of the Te ions away from their positions in the cubic lattice. The octahedra themselves are almost undistorted with all Te–Cl bonds measuring2.528(1) Å and a bond angle variance of 2.37 deg^2^.

### Crystal Structure of Low-Br MA_2_Te­(Cl_1–*x*_Br_*x*_)_6_

Single-crystal X-ray diffraction was carried out at 295 K on a low-Br MA_2_Te­(Cl_1–*x*_Br_*x*_)_6_ sample. The space group was determined to be *P*6_3_ *m*c, and the cell volume per formula unit was 335.3 Å^3^. As expected, this is larger than that in MA_2_TeCl_6_ due to the inclusion of some Br ions. The lattice parameter *a* increases to 7.3962(4) Å with the addition of Br, while *c* is doubled to 14.2588(14) Å, *Z* = 2 (*a* = 7.3565(4) Å and *c* = 7.0812(7) Å for MA_2_TeCl_6_).^6^ As illustrated in Figure 2(a), the structure follows the [TeX_6_]^2–^–MA^+^–MA^+^–[TeX_6_]^2–^ stacking sequence as MA_2_TeCl_6_ along the *c*-axis, and MA^+^ cations are still arranged parallel to the *c*-axis between octahedra. The [TeX_6_]^2–^ octahedra layer at *z* = 1/2 is separated from the layer at *z* = 0 by 2/3 and 1/3, respectively. For comparison, the octahedra in pure MA_2_TeCl_6_ are directly above each other along the *c-*axis.

**Figure 2:**
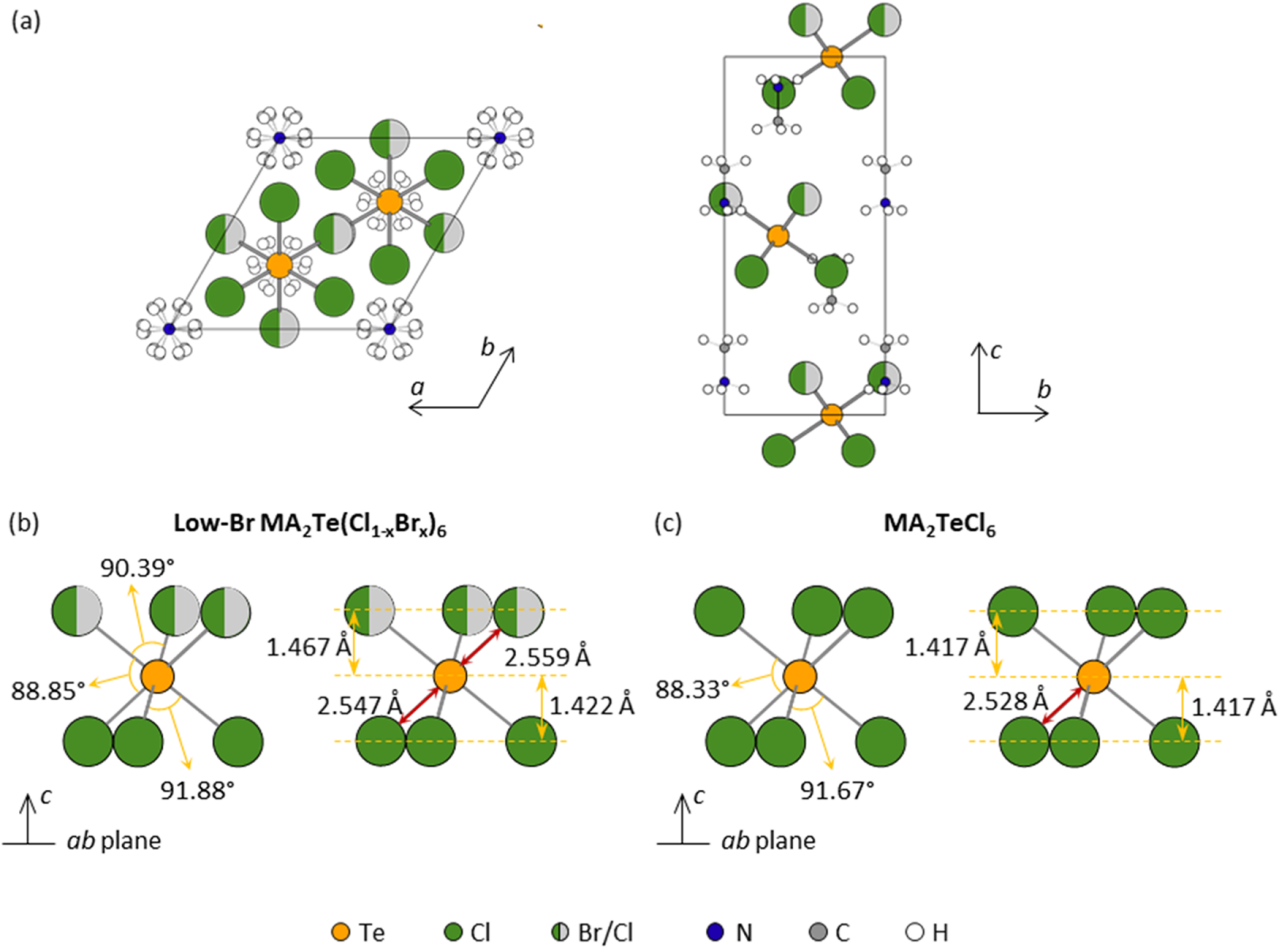
(a) Crystal structure of low-Br MA_2_Te­(Cl_1–*x*_Br_*x*_)_6_ at 295 K, viewed along the *c*-axis (left) and down the *a*-axis (right). Schematic diagram of the [TeX_6_]^2–^ octahedra in (b) low-Br MA_2_Te­(Cl_1–*x*_Br_*x*_)_6_ and (c) MA_2_TeCl_6_ in comparison. The values for MA_2_TeCl_6_ are obtained from previous work.^22^ The yellow, green, light gray, blue, dark gray, and white circles represent Te, Cl, Br, N, C, and H atoms, respectively.

The *P*6_3_ *m*c space group of the low-Br crystal is not a subgroup of *Fm*3̅*m* and cannot be reached from the cubic aristotype by distortion or rotation of the octahedra. The bromide ions were found to partially occupy three positions in each [TeX_6_]^2–^ octahedron (the X(1) site), which appeared equivalent to those obtained by SXD, indicating that their long-range average composition was the same. The remaining three positions are occupied only by chloride ions (X(2) site). The X(1) and X(2) positions thus form separate alternating layers parallel to the *ab* plane. Refinement shows 8.6% Br occupancy on the X(1) site, with no Br on the X(2) site.

This leads to the distortion of the octahedra, as depicted in Figure 2(b). Two Te–X distances are observed, and the Te–X(1) distance (2.559(3) Å) is slightly longer than the Te–X(2) distance (2.547(3) Å), as Br^–^ (1.96 Å) has a larger ionic radius than Cl^–^ (1.81 Å).^45^ The X(1)–Te–X(1) bond angle (90.39°) is smaller than the X(2)–Te–X(2) angle (91.88°). Consequently, the Te atom is not found equidistant between the X(1) and X(2) layers but is further away from the X(1) layer (1.467 Å) and closer to the X(2) layer (1.422 Å). The bond angle variance in the TeX_6_ octahedron is 1.72 deg^2^, which is less than that observed in MA_2_TeCl_6_(Figure 2(c)), where Te is symmetrically separated from the top and bottom Cl ions in a [TeCl_6_]^2–^ octahedron. The Cl atoms parallel to the *ab* plane have the same projected distances to the plane containing Te (1.417 Å) and bond angles (91.67°). All Te–Cl bond lengths are 2.547(3) Å at room temperature.

The projection distances along the *a*- and *c*-axes are summarized in Table 1. The distance between the [TeX_6_]^2–^ octahedra layers along the *c*-axis (**X­(1)**···**X­(2)**) is comparable to that of the pure MA_2_TeCl_6_ structure (**Cl**···**Cl**). Thus, the enlarged [TeX_6_]^2–^octahedra, due to Br-doping, are the main origin of the lattice expansion on *c*. On the other hand, the intra- and interoctahedra distances vary along the *a-*axis. Although the Te–X(1) bond length is lengthened by the addition of Br, the projected distances between halides along the *a*-axis may be identical to those in pure MA_2_TeCl_6_ due to the change in bond angles.

**Table 1:** Projected Bond Lengths (e.g., Cl–Cl) and Intermolecular Distances (e.g., Cl···Cl) Calculated Using Mercury^40^ ^a^

	Along the *a*-axis		Along the *c*-axis			
MA_2_TeCl_6_ *P*3̅*m*1	**Cl**···**Cl**	**Cl**–**Cl**	**Cl**···**Cl**	**Cl**–**Cl**	**N**···**Cl**	**C–N**
	3.730 Å	3.627 Å	4.248 Å	2.833 Å	0.136 Å	1.407 Å
Low-Br *P*6_3_ *mc*	**X(1)**···**X(1)**	**X(1)**–**X(1)**	**X(1)**···**X(2)**	**X(1)**–**X(2)**	**N(1)**···**X(1)**	**C(1)–N(1)**
	3.765 Å	3.632 Å	4.241 Å	2.889 Å	0.153 Å	1.356 Å
	**X(2)**···**X(2)**	**X(2)**–**X(2)**			**N(2)**···**X(2)**	**C(2)–N(2)**
	3.735 Å	3.661 Å			0.217 Å	1.359 Å
High-Br *R*3̅*m*	**X**···**X**	**X**–**X**	**X**···**X**	**X**–**X**	**N**···**X**	**C–N**
	3.760 Å	3.673 Å	4.220 Å	2.942 Å	0.252 Å	1.394 Å

a The values for MA_2_TeCl_6_ are obtained from previous work.^22^ Given that the typical error of the measured lattice parameters is less than 0.002 Å, the error for all of the projected distances and bond lengths is less than 0.002 Å.

### Crystal Structure of High-Br MA_2_Te­(Cl_1–*x*_Br_*x*_)_6_

At higher Br concentrations, the high-Br MA_2_Te­(Cl_1–*x*_Br_*x*_)_6_ at 295 K forms a rhombohedral structure *R*3̅*m*, which is isostructural to other methylammonium hexachlorometallates.^21^ *R*3̅*m* is a subgroup of *Fm*3̅*m*, and is reached from the aristotype by in-phase rotation of all octahedra by approximate 5° around the cubic axes.^46^ The cell volume is 342.7 Å^3^/formula unit, and the pseudocubic lattice parameter is 11.178(1) Å. As illustrated in Figure 3(a), the [TeX_6_]^2–^ octahedron layer at *z* = 1/3 is separated from the layer at *z* = 0 by 2/3 and 1/3, respectively.

**Figure 3:**
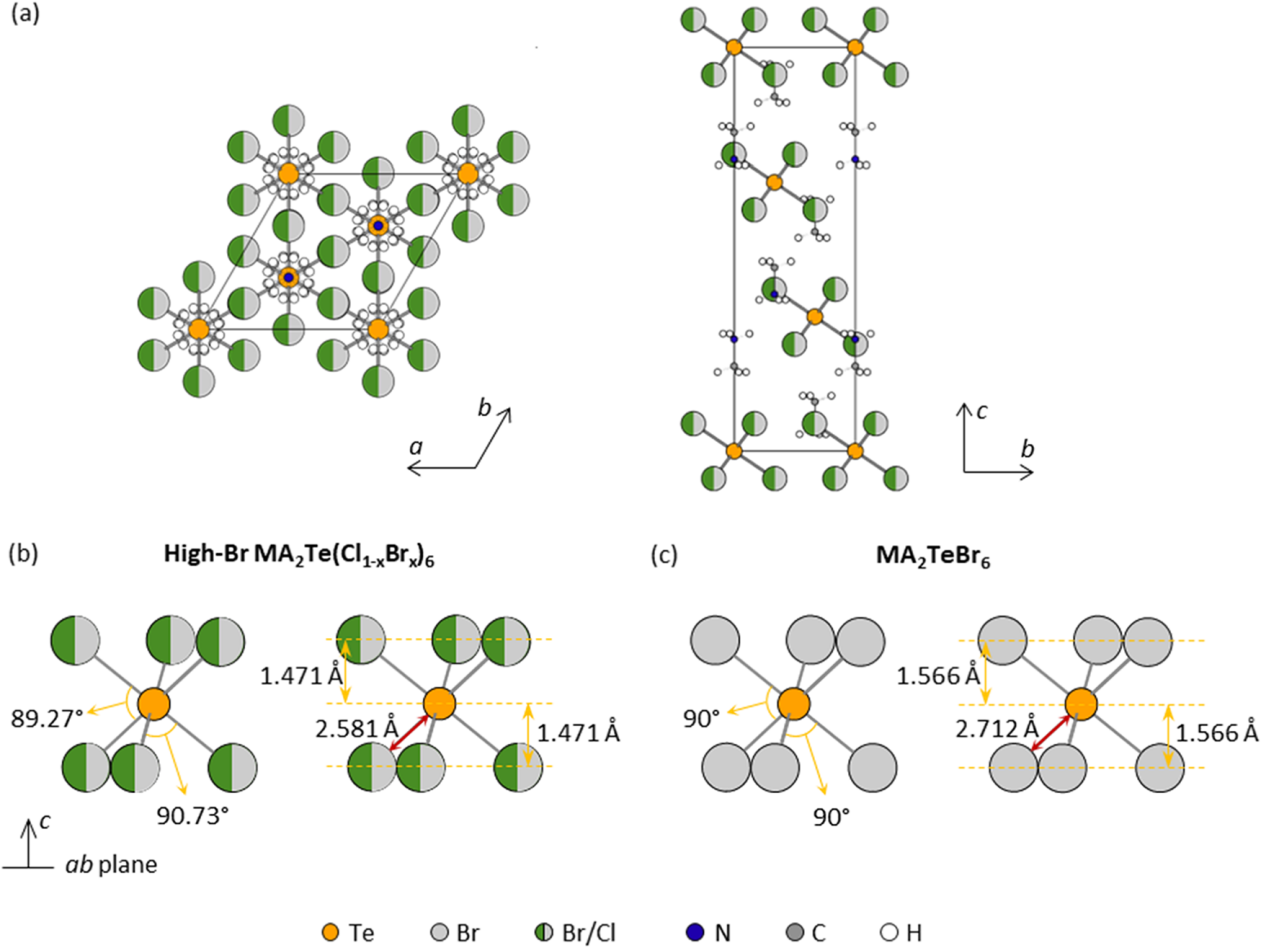
(a) Crystal structure of high-Br MA_2_Te­(Cl_1–*x*_Br_*x*_)_6_ at 295 K, viewed along the *c*-axis (left) and down the *a*-axis (right). Schematic diagram of the [TeX_6_]^2–^ octahedra in (b) high-Br MA_2_Te­(Cl_1–*x*_Br_*x*_)_6_ and (c) MA_2_TeBr_6_ in comparison. Yellow, green, light gray, blue, dark gray, and white circles represent Te, Cl, Br, N, C, and H atoms, respectively.

In the high-Br structure, no halide ordering is detectable, and all X positions have the same average Cl and Br occupancies. Refinement shows a 14.4% Br occupancy on the halide site, which matches well with the XPS compositional values (see Table S2). As a result, the bond lengths in [TeX_6_]^2–^ are identical at 2.581(1) Å. The bond angle variance of the octahedron is 0.53 deg^2^. Figure 3(b) depicts the octahedra in the high-Br structure. Te is symmetrically separated between the halide layers with a projected distance of 1.471 Å. The [TeBr_6_]^2–^ octahedron in MA_2_TeBr_6_ (Figure 3(c)) has a longer Te–Br bond length (2.712(2) Å) with a nondistorted structure. All bond angles are 90°.

The projection distances of the high-Br MA_2_Te­(Cl_1–*x*_Br_*x*_)_6_ lattice are summarized in Table 1. Although the octahedron is enlarged as the concentration of Br increases, the interoctahedral distances are shorter than those of MA_2_TeCl_6_. This is believed to be due to the different arrangements of the octahedral layers along the *c*-axis. However, the cation cavity is still expanded, which further increases the insertion of MA^+^ into the [TeX_6_]^2–^ layers, which will be further discussed later.

### Raman Spectroscopy

The discussion of the structure based on the SXD results indicates a partially ordered halide arrangement in the low-Br structure. As previous studies have shown a near-random halide distribution throughout the octahedra in mixed-halide compounds of the vacancy-ordered double perovskites,^35,47^ the low-Br structure reported in this work appears unusual. Raman spectroscopy can be an effective local probe to understand the halide distribution in the compound. This is due to the presence of isolated octahedra in the double perovskites.

Following the work of Karim et al.,^35^ DFT was used to simulate the Raman-active vibrational frequencies of Te octahedra with various Cl and Br octahedral coordinations. Since the Raman spectra of compounds with this structure type are dominated by the vibrational modes of the isolated [BX_6_]^2–^ octahedra, it is sufficient to calculate the Raman spectrum from each possible octahedron and compare it with the experimental data. Experimental Raman spectra were collected for comparison with the calculated spectra. This method was first validated using pure halide perovskites, MA_2_TeCl_6_ and MA_2_TeBr_6_. Figure S6 shows a good match between the experimental and calculated Raman spectra. The three Raman-active peaks correspond to the A_1g_, E_g_, and T_2g_ vibrations of a regular octahedron.

The possible local environments around Te in the mixed-halide compounds were then simulated by substituting an increasing number of Br ions on the 6 possible coordination sites in the primitive structure. Eight mixed-anion coordinations are possible (see Figure S7): [TeCl_5_Br]^2–^, *cis*-[TeCl_4_Br_2_]^2–^, *trans*-[TeCl_4_Br_2_]^2–^, *mer*-[TeCl_3_Br_3_]^2–^, *fac*-[TeCl_3_Br_3_]^2–^, *cis*-[TeCl_2_Br_4_]^2–^, *trans*-[TeCl_2_Br_4_]^2–^, and [TeClBr_5_]^2–^. The experimental Raman spectra for both low- and high-Br appear very similar, as shown in Figure S8, indicating that they have similar [TeX_6_]^2–^ coordination. In addition to the three peaks observed originating from [TeCl_6_]^2–^, there is an additional peak at *ca*. 190 cm^–1^ for both crystals, which has a higher intensity in the high-Br sample. Hence, it is reasonable to assume that both low-Br and high-Br samples contain the same type of [TeX_6_]^2–^, while the latter has a greater concentration of the octahedron responsible for the 190 cm^-1^ peak..

Figure 4 shows a comparison between the various DFT-calculated vibrational modes (in blue) and the high-Br experimental spectrum (in yellow). The best match to the experimental spectrum is observed with the calculated spectrum for [TeCl_5_Br]^2–^, and all others have a different number of vibrational modes and/or relative positions of the modes. Therefore, the Raman analysis suggests the homogeneity of octahedra that there is at most one Br atom within each [TeX_6_]^2–^ octahedron in both low- and high-Br structures.

**Figure 4:**
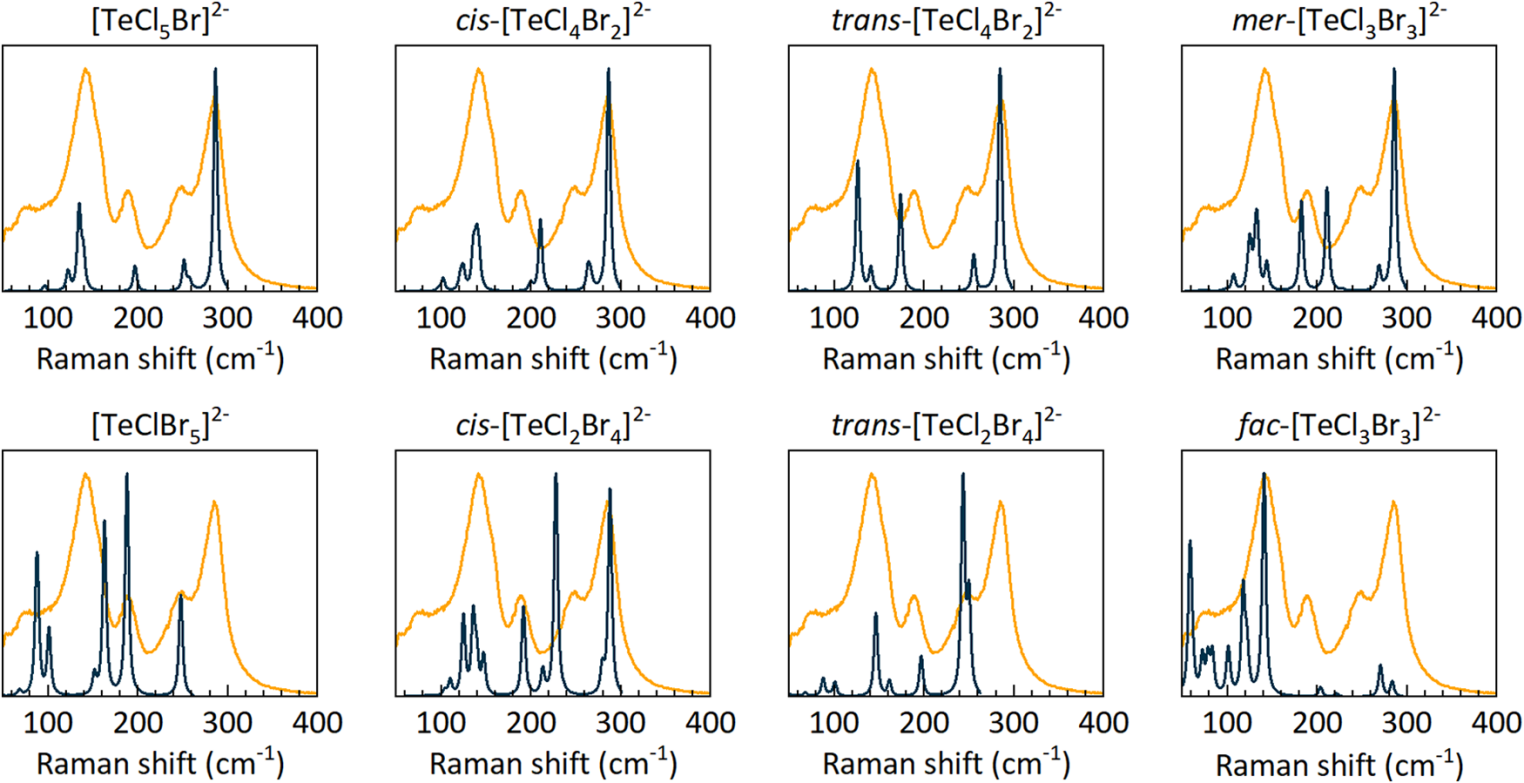
Comparison between the experimental Raman spectra (yellow) of high-Br MA_2_Te­(Cl_1–*x*_Br_*x*_)_6_ and DFT-calculated spectra (blue) for different types of [TeCl_6‑x_Br_*x*_]^2–^ octahedra.

SXD refinement places this Br ion in the X(1) plane, and the rest is occupied by Cl in the low-Br phase. If every octahedron contained exactly one Br ion, the Br anion content would be $\frac{1}{6}$ or 16.7%. In fact, the low-Br sample with ordered anions has only 6.8% Br from XPS, which would correspond to around 1 in 3 octahedra having a Br ion in the X(1) plane. The refined average occupancy of the X(1) site is 8.6% Br, which is close to 1 in 2 of the octahedra having a Br ion in the X(1) plane. The ordering observed in the low-Br compound may be dependent on the precise size of the octahedra relative to the A-site ion, which stabilizes the *P*6_3_ *m*c structure.

Turning to the high-Br structure, which shows no anion ordering by SXD, the Raman also leads us to a similar conclusion that, aside from [TeCl_6_]^2–^, the only other octahedron present in significant amounts is [TeCl_5_Br]^2–^. However, the higher concentration of Br in the high-Br compound (10.1% from XPS, 14.4% from SXD refinement) indicates that more than half of the octahedra contain a Br ion in this compound. The reason for the lack of anion ordering in this composition is not clear; however, we can draw some conclusions from the solution-synthesized powder sample results. As shown in the SI, the nominal 6% Br sample contains 15.7% Br based on the XPS results, while its PXD pattern (Figure S1) primarily consists of a low-Br structure, with a minor component of a high-Br phase. The Raman spectra (Figure S9) matched well with the calculated spectrum for [TeCl_5_Br]^2–^. When the Br concentration further increases (>16.7%), a clear difference is observed in the Raman spectra. The peaks at *ca*. 73 and 156 cm^–1^ are consistent with the calculated spectrum for *fac*-[TeCl_3_Br_3_]^2–^, and their increased intensity as well as the diminishing intensity above 200 cm^–1^ provide further evidence for the increased formation of *fac*-[TeCl_3_Br_3_]^2–^. This does not imply that the octahedra in these compounds comprise solely [TeCl_6_]^2–^ , [TeCl_5_Br]^2–^ and *fac*-[TeCl_3_Br_3_]^2–^; rather, other components cannot be identified clearly.

Although the underlying cause of the polar ordering in the narrow composition range is not fully understood, we suggest some important factors. In the polar low-Br compound, the octahedra present are [TeCl_6_]^2–^ and [TeCl_5_Br]^2–^. The preference for [TeCl_5_Br]^2–^ over other mixed-anion octahedra is influenced by the overall halide composition. With the low-Br content making the presence of two or more Br ions in a single octahedron less likely, it is possible that this may also be driven by the greater stability of [TeCl_5_Br]^2–^. Thus, the preference for this specific octahedron may have an electronic basis. The second requirement for the polar phase is the arrangement of [TeCl_5_Br]^2–^ octahedra such that all Br ions fall in a common layer. This is likely driven by size effects, with the observed polar structure being an ordered arrangement available at this specific concentration of [TeCl_5_Br]^2–^. Increasing the concentration of [TeCl_5_Br]^2–^, as in the high-Br sample, or introducing other octahedra, such as in the solid-state synthesized compounds (as discussed in the SI), prevents the formation of an ordered polar structure. These collections of octahedra evidently do not order in the same way as the low-Br sample.

Partial anion ordering in low-Br MA_2_Te­(Cl_1–*x*_Br_*x*_)_6_ leads to a polar structure that can exhibit piezoelectric properties. Materials that can be classified as piezoelectric are restricted to noncentrosymmetric point groups where the primitive unit cells possess a nonvanishing dipole moment. In the double perovskite, the interplay between the dynamics of the molecular ion (MA) and the inorganic octahedral cage leads to a noncentrosymmetric crystal structure.^48,49^ *P*6_3_ *mc* is a space group, which is known to be piezoelectric.^50–52^ When external forces are applied, the unit cell experiences polarization and is able to sustain a dipole moment. An example is the double-perovskite K_2_MnF_6_ that crystallizes in both *Fm*3̅*m* (*c*) and *P*6_3_ *mc* (*h*) space groups, but only presents piezoelectricity as h*-*K_2_MnF_6_, as it is symmetry-forbidden in its cubic phase.^51^

To attempt to measure the piezoelectric response, PFM was performed on the single-crystal surfaces, as illustrated in Figure 5. An AC electric field was applied to the sample while the AFM tip was maintained with a constant deflection over the sample. In response to the applied AC field, a piezoelectric material can expand or contract, resulting in a change in the deflection of the cantilever. Figure 5(a–c) shows the representative tomography of the crystal surface over a 10 × 10 μm^2^ area, with the corresponding piezoresponse amplitude under an applied sample bias of 3 V shown below. Tomography shows light damage to the crystal surface, possibly due to the tweezers’ handling. The piezoresponse is measured as a deflection error to detect the presence of lateral piezoelectric domains.

**Figure 5:**
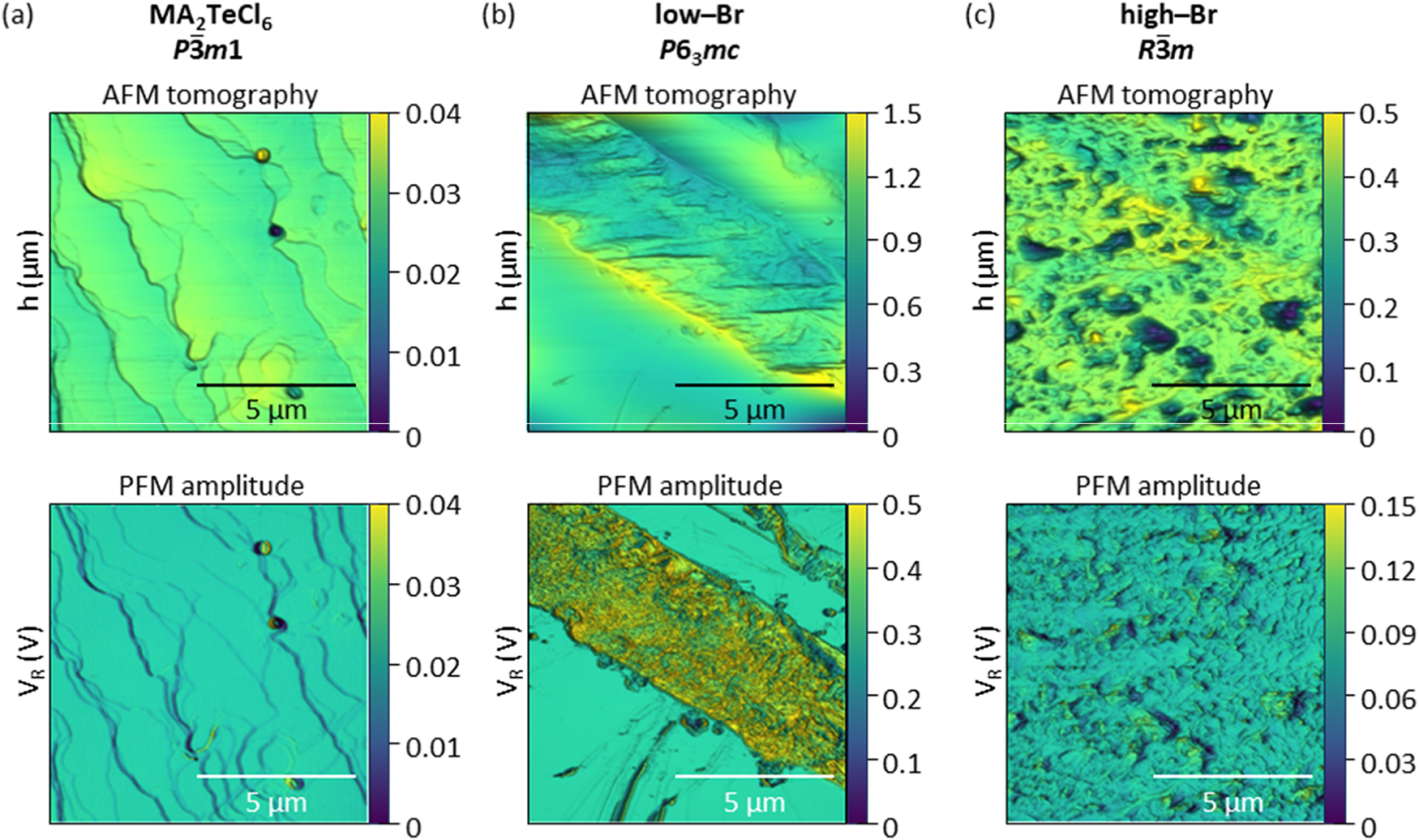
Topography and PFM signal on the (a) MA_2_TeCl_6_, (b) low- and (c) high-Br MA_2_Te­(Cl_1–*x*_Br_*x*_)_6_ single-crystal surfaces over a 10 × 10 μm^2^ area.

For the low-Br MA_2_Te­(Cl_1–*x*_Br_*x*_)_6_ in Figure 5(b), a noticeable difference in the piezoresponse amplitude reveals the domain pattern. The presence of yellow and blue-green regions corresponds to low and high PFM amplitudes, respectively. The difference between the regions indicates the presence of specific polarization orientations within the compound: yellow is in-phase and the blue-green is out-of-phase to the incident electric field, which is beyond the scope of this work.^53,54^ Meanwhile, the piezoresponse was absent for pure MA_2_TeCl_6_ (Figure 5(a)), while significantly lower and scattered for the high-Br phase (Figure 5(c)). These observations indicate that the presence of Br ions in the [TeX_6_]^2–^ octahedron does not automatically result in a piezoelectric structure. However, the Br/Cl ordering along the *c*-axis leads to a noncentrosymmetric crystal structure, which is essential to retain a dipole moment. The lattice distortions from the AC field result in polarization and observable piezoresponses.^55,56^ Thus, the PFM results further support the presence of a noncentrosymmetric structure of low-Br MA_2_Te­(Cl_1–*x*_Br_*x*_)_6_.

### A-Site Cation Cavity

The A cation cavity undergoes alteration as the structure changes across the composition range studied. Although in each case the A-site cavity is still formed by 12 halide neighbors, its coordination changes with the different crystal structures, as depicted in Figure 6. We have previously determined that the MA^+^ cation adopts a conical rotation in MA_2_TeCl_6_,^22^ and we now carry out similar refinements of the average MA^+^ position for the other structures reported here.

**Figure 6:**
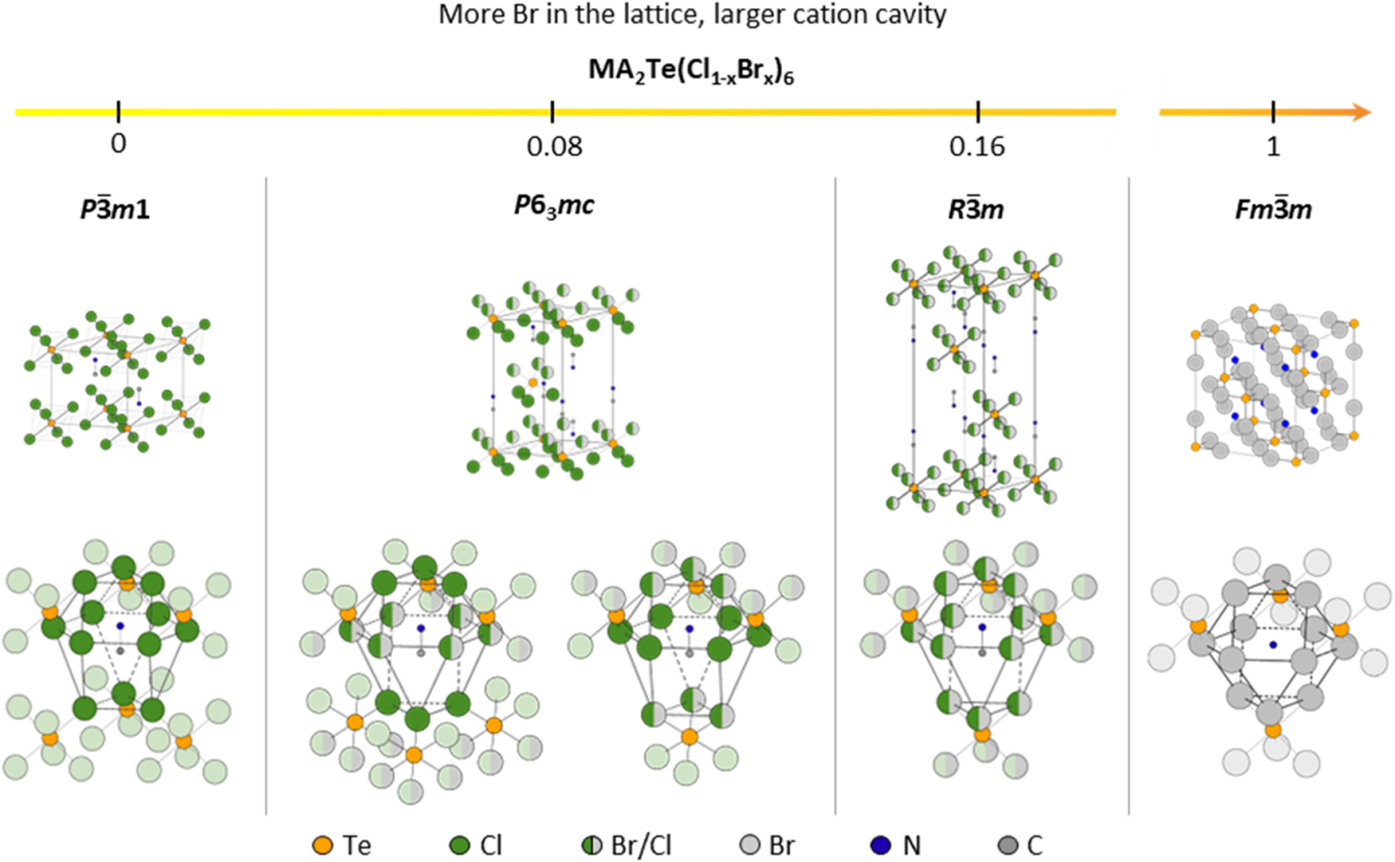
Schematic diagram of the unit cells (top) and MA cations located in the cavities (bottom) in MA_2_TeCl_6_, low-Br MA_2_Te­(Cl_1–*x*_Br_*x*_)_6_, high-Br MA_2_Te­(Cl_1–*x*_Br_*x*_)_6_, and MA_2_TeBr_6_ at room temperature. For the sake of clarity, the hydrogen atoms are not shown. The MA cation is replaced by one N atom in MA_2_TeBr_6_ to represent the spherical rotation of MA^+^.

Uniquely for the compounds studied here, the low-Br structure with partial anion ordering has two distinct A-site environments: one of these (Figure 6(b), left) is similar to that found in MA_2_TeCl_6_, where MA^+^ is coordinated to six [TeX_6_]^2–^ anions, with the bottom octahedra layer rotated by 180°. The other A-site environment (Figure 6(b), right) has a structure similar to that found in high-Br MA_2_Te­(Cl_1–*x*_Br_*x*_)_6_ and MA_2_TeBr_6_, as shown in Figure 6(c,d), respectively, with the A-site surrounded by four [TeX_6_]^2–^ anions. In this way, the low-Br structure can be thought of as a mixture of the environments observed in MA_2_TeCl_6_ and MA_2_TeBr_6_.

Although the interlayer distance of the [TeX_6_]^2–^ octahedra (**X­(1)**···**X­(2)**) remains similar in the low-Br structure to that of MA_2_TeCl_6_, the A-site cavity itself is slightly larger due to the increased size of the octahedra. The MA^+^ cations in the low-Br phase are ‘inserted’ more into the [TeX_6_]^2–^ layer, as indicated by the increased projected **N­(1)**···**X­(1)** and **N­(2)**···**X­(2)** distances in Table 1. The MA^+^ cations surrounded by their cation cavities are illustrated in Figure S10. Both types of cavities in the low-Br structure contain, on average, 6 Cl and 6 Br ions; however, they have different halide coordinations. Therefore, although the heights of the two tetradecahedra and the hexagonal areas of their middle layers are almost the same, their volumes vary due to the difference in the length (i.e., **X­(1)**···**X­(1)** and **X­(2)**···**X­(2)**) of their top and bottom sides. The space near N(1) is smaller than that near N(2), which allows N(2) to insert deeper into the [TeX_6_]^2–^ layer, with a larger projected distance (**N**···**X**). Different halide coordinations also affect the molecular dynamics of MA^+^. As illustrated by the ellipsoids in Figure S10­(a), the N(1) spins have a more ellipsoidal shape, while N(2) is more spherical. This could explain the slightly increased C–N(2) bond length (1.359 Å) compared to C–N(1) (1.356 Å).

The MA^+^ cation in high-Br MA_2_Te­(Cl_1–*x*_Br_*x*_)_6_ is coordinated to four [TeX_6_]^2–^ octahedra, similar to the observed A-site cavity in MA_2_SnCl_6_.^57^ The cavity size increases with additional Br (Figure S10­(b)), resulting in further penetration of MA into the Te halide layers. The smaller distances between N and X may restrict the conical rotation of MA^+^, hence increasing the projected C–N length on *c* to 1.394 Å, which is closer to the bond length in methylamine (1.469 Å).^58^ As the Br concentration further increases to MA_2_TeBr_6_ (Figure 6(d)), the cavity shares a similar but now more symmetric tetradecahedron structure. The cavity space is sufficiently enlarged to allow for the spherical rotation of the MA molecule.

Overall, the MA^+^ dynamics are closely related to the cavity size and shape. The rotation mode can be interpreted from the projected C–N bond length: MA^+^ first experiences a more ‘conical’ rotation from MA_2_TeCl_6_ to low-Br structure, and then it is allowed to stay more vertically in the high-Br sample with a larger interlayer distance of [TeX_6_]^2–^ octahedra. Finally, with a much larger cavity, MA^+^ rotates in a spherical manner in MA_2_TeBr_6_.

## Conclusion

The counter-diffusion crystal growth technique is advantageous due to its ability to facilitate precise control over the halide ratio incorporated into the crystal lattice. Hence, low- and high-Br MA_2_Te­(Cl_1–*x*_Br_*x*_)_6_ single crystals have been synthesized using the CDCG technique. Phase transitions are clearly observed by increasing the Br concentration in the MA_2_Te­(Cl_1–*x*_Br_*x*_)_6_ (0 ≤ *x* ≤ 1) series at room temperature. The structure of low-Br MA_2_Te­(Cl_1–*x*_Br_*x*_)_6_ is identified as *P*6_3_ *mc*, consistent with its piezoelectric response at room temperature, while the high-Br MA_2_Te­(Cl_1–*x*_Br_*x*_)_6_ shares the same nonpiezoelectric crystal structure, *R*3̅*m*, as MA_2_SnCl_6_. The structural evolution and molecular dynamics of MA_2_Te­(Cl_1–*x*_Br_*x*_)_6_ were analyzed in detail. Uniquely, the halide distribution within the two mixed-halide compounds appears to be ordered with at most one bromine atom per [TeX_6_]^2–^ octahedron. This feature is distinct from the more common statistical or random mixing observed in many other mixed-halide perovskites.

## Supplementary Material



## Notes

Additional results on solid state and solution phase syntheses, and Raman calculations (PDF)
