# Supplementary material for: Observation of a Polar Compound with Halide Ordering in the Compositional Series (CH3NH3)2Te(Br x Cl1–x )6
Source: Inorg Chem. 2025 Oct 22;64(43):21479–88. doi: 10.1021/acs.inorgchem.5c03380 (PMC12587395; doi:10.1021/acs.inorgchem.5c03380)
Supplement: Supplementary file 1 [file ic5c03380_si_001.pdf]

Supporting information

Observation of a Polar Compound with Halide Ordering in the Compositional Series $(CH_3NH_3)_2Te(Br_xCl_{1-x})_6$

Yuhan Liu[1,2], Prajna Bhatt[1,3], Roxy Lee[1,4], Avishek Dey[1,5], Anna Regoutz[1,6], Robin S. Perry[5,7], Robert G. Palgrave*[1]

[1] Department of Chemistry, University College London, 20 Gordon St, London, WC1H 0AJ, UK
[2] Department of Chemical Engineering, University College London, Torrington Place, London, WC1E 7JE, UK
[3] Istituto Officina dei Materiali (IOM)-CNR, Laboratorio TASC, in Area Science Park, S.S.14, Km 163.5, Trieste I-34149, Italy
[4] Department of Energy Conversion and Storage, Danmarks Tekniske Universitet, Fysikvej, 310, 334 2800 Kgs. Lyngby, Denmark
[5] London Centre for Nanotechnology, University College London, London, WC1H 0AH, UK
[6] Department of Chemistry, University of Oxford, Inorganic Chemistry Laboratory, South Parks Road, OX1 3QR, Oxford, UK
[7] Department of Physics and Astronomy, University College London, London WC1E 6BT, United Kingdom

Table of Contents

Synthesis of $MA_2Te(Cl_{1-x}Br_x)_6$ from solution precipitation 2

Comparison of $MA_2Te(Cl_{1-x}Br_x)_6$ synthesised from different methods 4

Raman spectra of $MA_2Te(Cl_{1-x}Br_x)_6$ 7

Synthesis of $MA_2Te(Cl_{1-x}Br_x)_6$ powder from solution precipitation

The structures in the $MA_2Te(Cl_{1-x}Br_x)_6$ series was initially explored with powder synthesis using solution precipitation methods. TeO_2 was reacted with a stoichiometric amount of mixed hydrohalic acid (37 wt.% HCl and 47–49 wt.% HBr from Sigma Aldrich), and methylamine solution (Sigma Aldrich, 40 wt.%) were reacted with HCl individually. Then the resulting TeX_4 (X = Br, Cl) solution was added into methylamine chloride (MACl) solution at room temperature. Precipitates were formed immediately, the mixture was then filtered, washed with diethyl ether and dried at 60 °C to obtain MA_2TeX_6 powders.

The PXD pattern of the whole series are shown in Figure S1 with nominal concentrations, and the measured compositions from XPS are shown in Table S1. The PXD patterns from these samples can be interpreted using the single crystal structures discussed in the main paper. The pure cubic phase is observed at 100% and 87% Br, with decreasing lattice parameter as the Br concentration falls. At 60.9% Br, the $R\bar{3}m$ phase is observed in addition to the cubic phase, indicating the phase transition is close to this composition. The $R\bar{3}m$ phase is observed down to 19.8% Br, while the polar $P6_3mc$ phase is seen at 15.7% Br. Lower Br concentrations give the $P\bar{3}m1$ structure shared with MA_2TeCl_6. Since the crystal structures could not be solved from the PXD data, this motivated us to try single crystal growth within 4.8-19.8% Br where the new phases were observed.

Table S1 Atomic ratio of solution-synthesized $MA_2Te(Cl_{1-x}Br_x)_6$ powder. Quantification was done using the Cl 2*p*, Br 3*d* core levels spectra in Thermo Avantage.

**Sample (nominal %Br)**	$\frac{[Br]}{[Br]+[Cl]}$ **ratio (XPS)**	**Phases present from PXD**
MA_2TeCl_6	0%	$P\bar{3}m1$
MA_2TeCl_6 4%Br	4.8%	$P\bar{3}m1$
MA_2TeCl_6 6%Br	15.7%	$P6_3mc$
MA_2TeCl_6 10%Br	19.8%	$R\bar{3}m$
MA_2TeCl_6 20%Br	23.9%	$R\bar{3}m$
MA_2TeCl_6 30%Br	38.0%	$R\bar{3}m$
MA_2TeCl_6 40%Br	50.2%	$R\bar{3}m$
MA_2TeCl_6 50%Br	60.9%	$Fm\bar{3}m$ + $R\bar{3}m$
MA_2TeCl_6 90%Br	87.0%	$Fm\bar{3}m$
MA_2TeBr_6	100%	$Fm\bar{3}m$

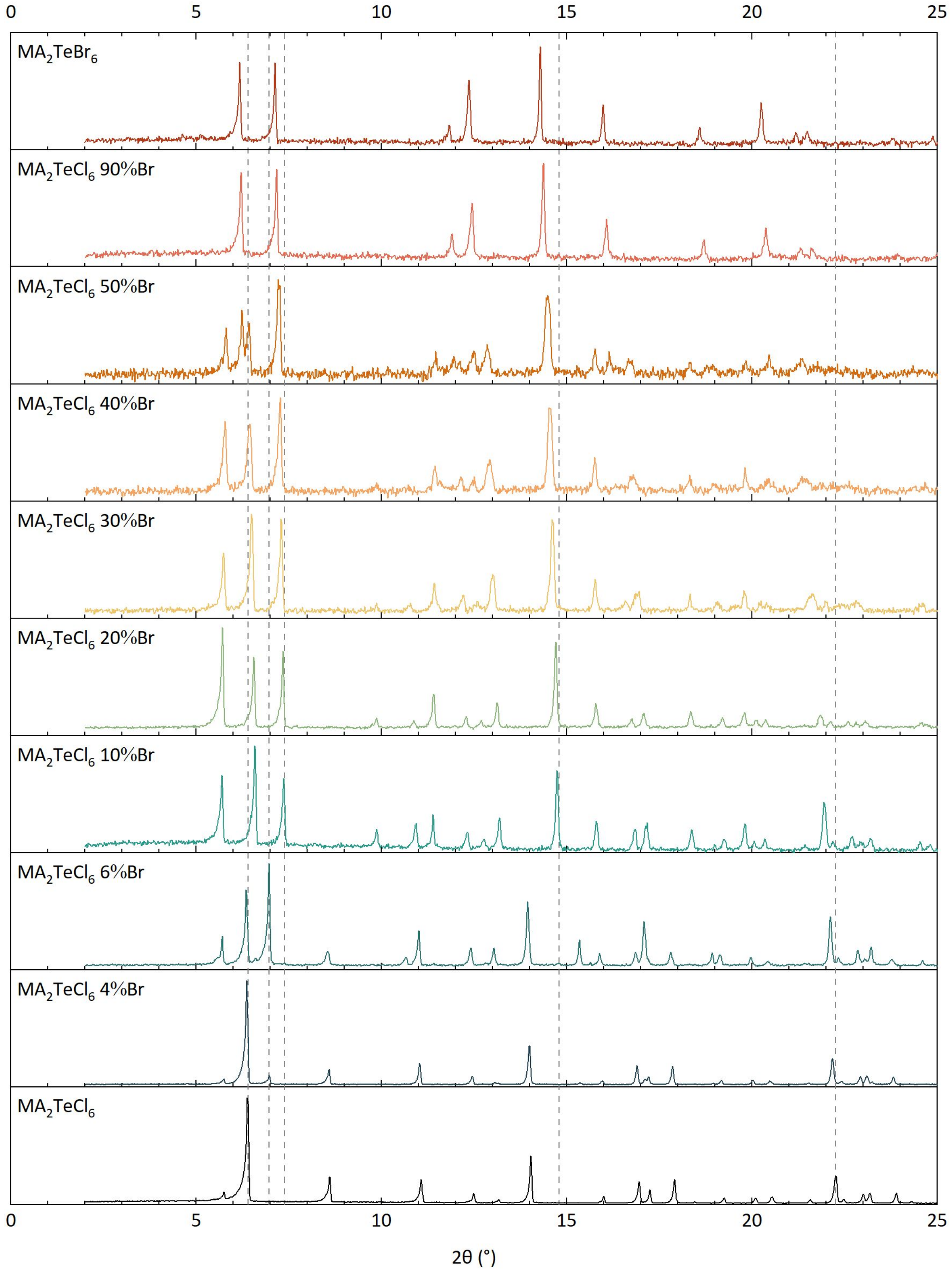

Figure S1 PXD patterns of solution-synthesized $MA_2Te(Cl_{1-x}Br_x)_6$ powder, collected with Mo radiation ($K\alpha_1$ with λ = 0.7093 Å). Peak shifts to lower angle can be observed with the concentration of bromide increases. All patterns are labelled with nominal Br concentrations.

Comparison of $MA_2Te(Cl_{1-x}Br_x)_6$ synthesised using different methods

Synthesis of the $MA_2Te(Cl_{1-x}Br_x)_6$ was also attempted high temperature solid state methods. Solid state sintering involved mixing pure MA_2TeCl_6 and MA_2TeBr_6 together in different proportions. These were subsequently ground by hand in agate mortar and pestle then sealed in quartz ampoules under vacuum (c. 10^{-3} mbar), then heated to 150 °C or 180 °C for 2 hours. When heated above 180 °C, the powder colour darkened and subsequent PXD indicated decomposition had occurred.

Figure S2 compares the attempted synthesis of the low–Br compound obtained from different synthesis methods, and for comparison the CDCG single crystals of the high–Br phase. Both solid state and solution syntheses result in a mixture of the low- and high-Br phases. Asterisks represent peaks arising from the remaining precursors MA_2TeCl_6 and MA_2TeBr_6, which can be reduced by increasing the sintering time (Figure S3). However, 36 h sintering was insufficient to obtain a pure phase. In addition, although the Br/Cl ratio was controlled to be 8.3% for low–Br $MA_2Te(Cl_{1-x}Br_x)_6$, Table S2 showing that the obtained halide ratio measured by XPS varies. Achieving precise control over mixed halide composition is a known difficulty in solution processing.

CDCG, however, showed the best capacity for compositional control, possibly due to the slower crystal growth compared to other methods. Traditional solution-based crystal growth methods, such as utilizing solvent evaporation or temperature gradient to induce a high degree of supersaturation, can lead to rapid, uncontrolled nucleation. Subsequently, the formation of numerous small crystallites will contain a high density of defects. In contrast, the use of a silica gel in CDCG creates a porous, viscous medium that limits the ion mobility and eliminates convection. This slow, diffusion-controlled environment ensures a homogeneous distribution of bromine atoms throughout the crystal formation process, which is crucial for stabilizing the ordered structure. We believe this process is kinetically driven. This is supported by the failure of solid-state synthesis to produce low– and high–Br phases indicating thermodynamic equilibrium does not favour this halide-ordered structure. Additionally, in CDCG the synthesis of low- and high-Br phases were reproducible with the same nominal Br/Cl ratio and experimental procedure.

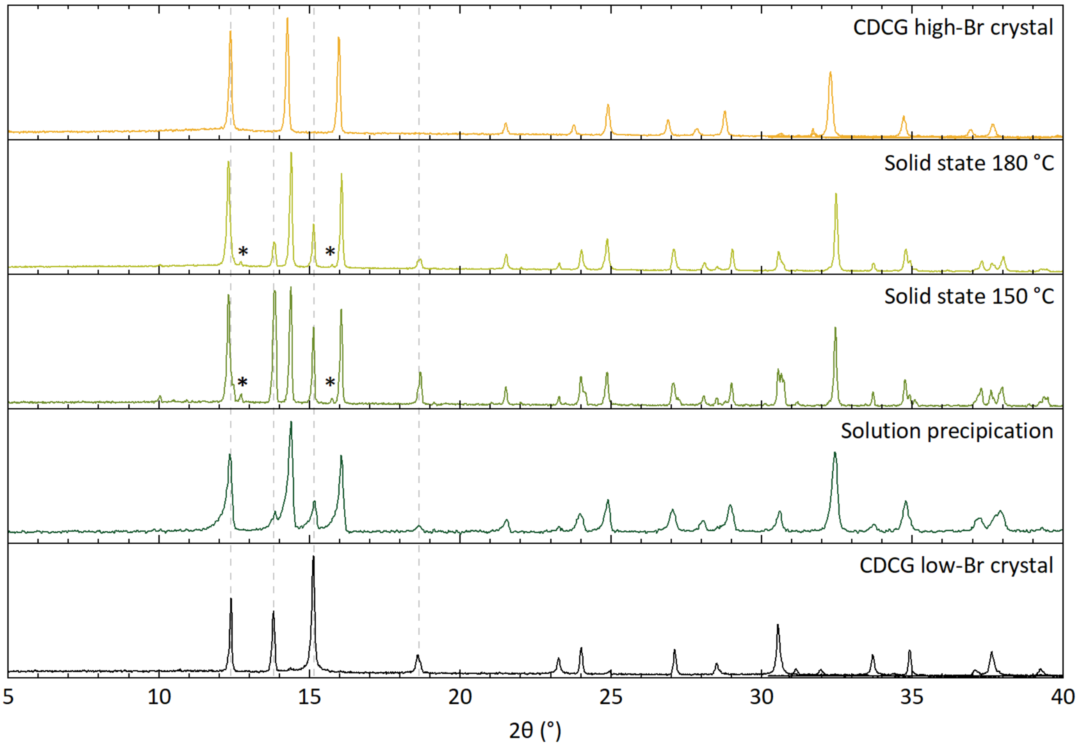

Figure S2 PXD patterns of the $MA_2Te(Cl_{1-x}Br_x)_6$ samples synthesised via different approaches. Asterisks represent the remaining starting materials. Dashed grey lines mark the low–Br $MA_2Te(Cl_{1-x}Br_x)_6$ diffraction peaks between 10 and 20°.

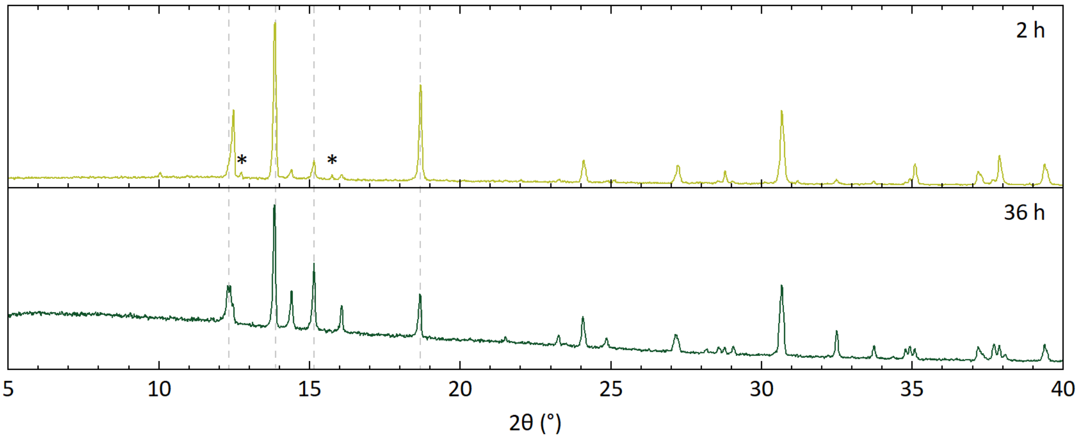

Figure S3 PXD patterns of the $MA_2Te(Cl_{0.96}Br_{0.04})_6$ at 150 °C for 2 h and 36 h. Asterisks represent the remaining starting materials. Dashed grey lines mark the low–Br $MA_2Te(Cl_{1-x}Br_x)_6$ diffraction peaks between 10 and 20°.

Table S2 Atomic ratio of low–Br and high–Br $MA_2Te(Cl_{1-x}Br_x)$ samples synthesised via different approaches. Quantification was done using the N 1*s*, Te 3*d*, Cl 2*p*, Br 3*d* core levels spectra in Thermo Avantage.

	**Synthesis Method**	**XPS** $\frac{[Br]}{[Br]+[Cl]}$ **ratio**
**low–Br**	Solid state 180°C	9.7%
	Solid state 150°C	11.1%
	Solution precipitation	15.7%
	**CDCG**	6.8%
**high–Br**	**CDCG**	10.1%

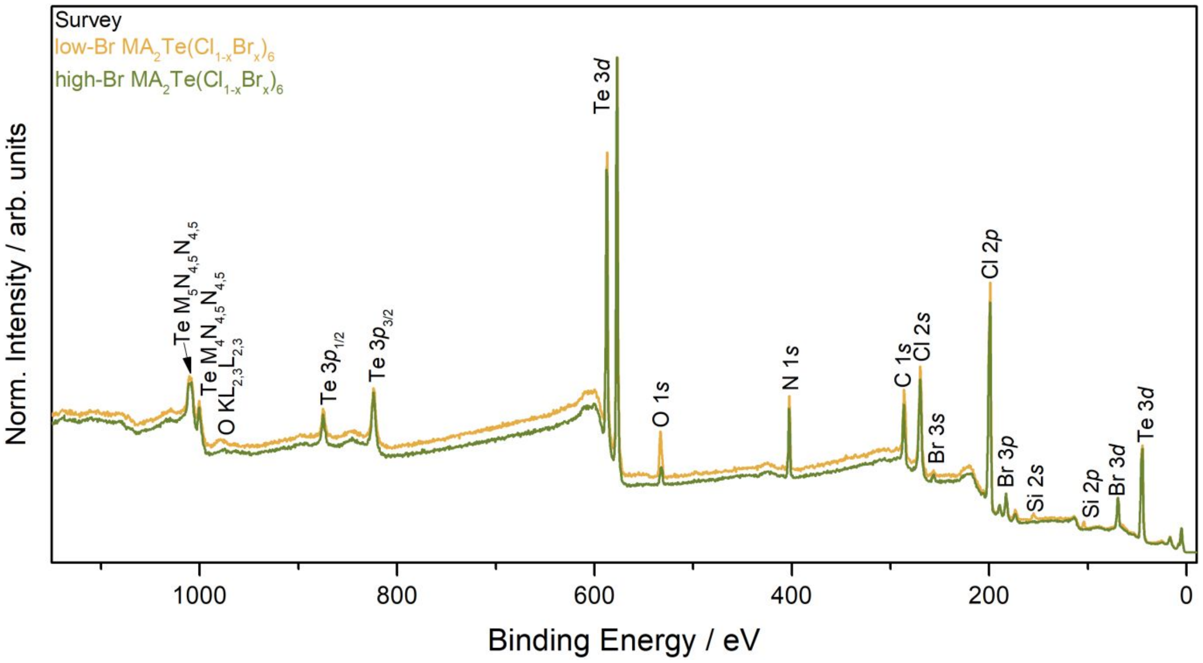

Figure S4 X-ray photoelectron survey spectra of low– and high–Br $MA_2Te(Cl_{1-x}Br_x)_6$ crystal surfaces produced by CDCG. Data were normalized to the height of the Te 3*d* peak.

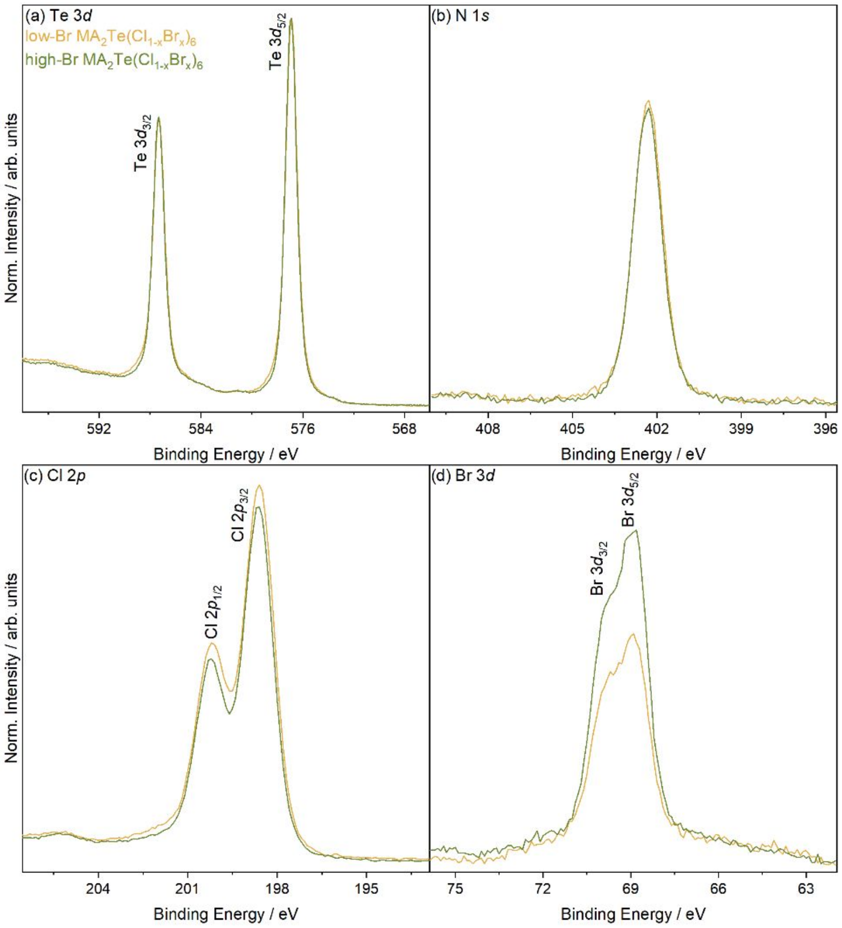

Figure S5 X-ray photoelectron core level spectra of low– and high–Br $MA_2Te(Cl_{1-x}Br_x)_6$ crystals produced by CDCG, including (a) Te 3*d*, (b) N 1*s*, (c) Cl 2*p* and (d) Br 3*d*. Data were normalized to the height of the Te $3d_{5/2}$ peak after a linear background removal.

Raman spectra of $MA_2Te(Cl_{1-x}Br_x)_6$

The Raman active vibrational frequencies and their expected intensities from $[TeCl_6]^{2-}$ and $[TeBr_6]^{2-}$ were calculated using DFT. According to the DFT calculation, the vibrations of $[TeCl_6]^{2-}$ have 126, 235, and 279 cm^{-1} frequencies, while those of $[TeBr_6]^{2-}$ have 81, 146 and 170 cm^{-1} frequencies. Three Raman active modes at 138, 248, and 294 cm^{-1} were recorded for MA_2TeCl_6, and 67, 144, and 165 cm^{-1} for MA_2TeBr_6, which are assigned as the F_{2g}, E_g, and A_{1g} vibrations of the octahedron, respectively.

The differences between predicted and experimental vibrational frequencies are probably due to the lack of finite temperature effects in the calculation.[35] Thus after peak shift and temperature correction is applied to the calculated frequencies, the calculated spectra match well with experimental data as shown in Figure S6. Therefore, this method has been validated.

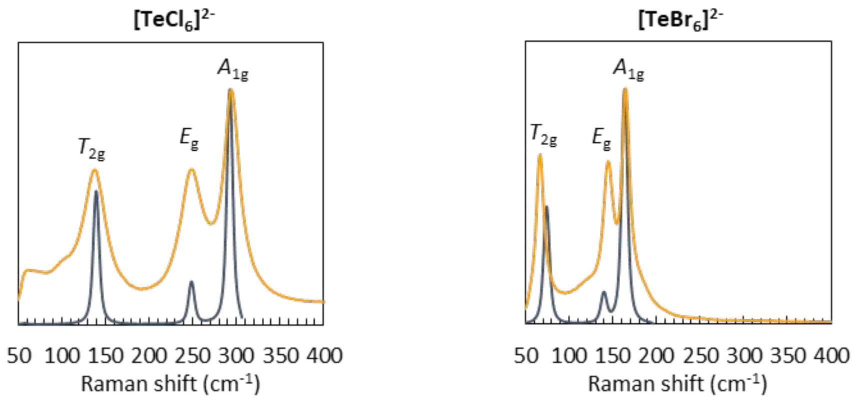

Figure S6 Representative experimental Raman spectra (yellow) and calculated Raman spectra (blue) of MA_2TeCl_6 and MA_2TeBr_6, normalized to the A_{1g} peaks.

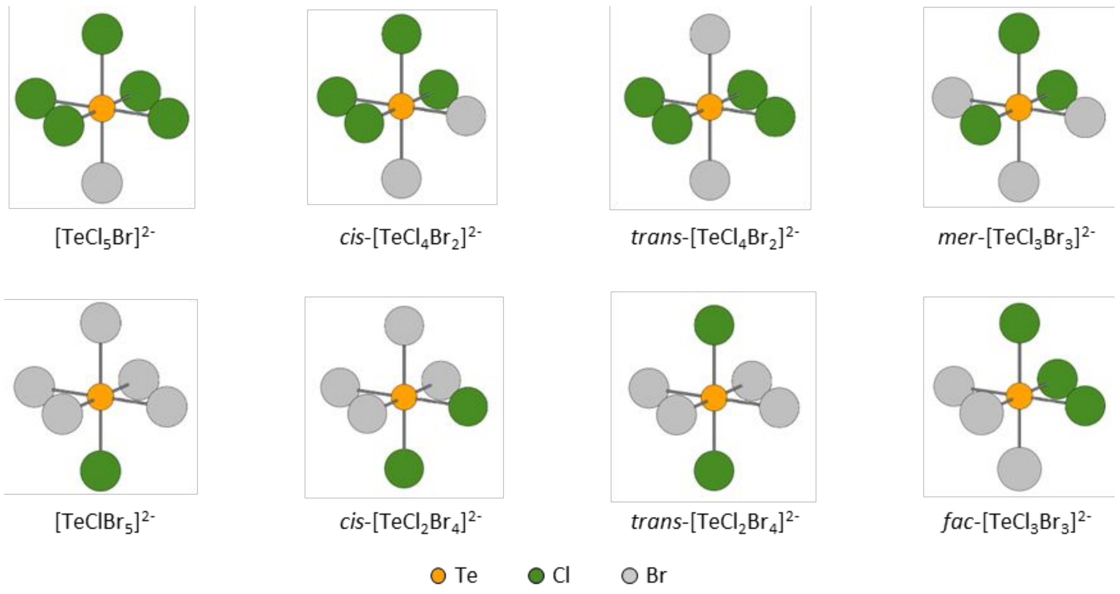

Figure S7 Schematic diagram of different $[TeCl_{6-x}Br_x]^{2-}$ octahedra.

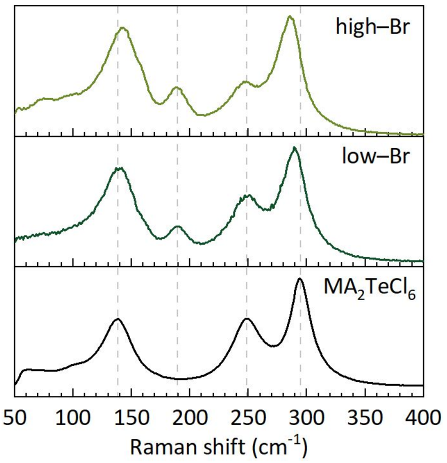

Figure S8 Representative experimental Raman spectra of CDCG single crystal samples comparing to pure MA_2TeCl_6.

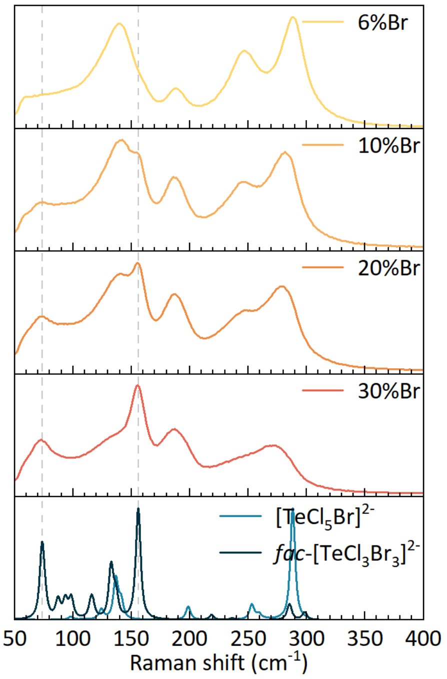

Figure S9 Representative experimental Raman spectra of nominal MA_2TeCl_6 6%, 20% and 30%Br powder samples, compared to calculated $[TeCl_5Br]^2$ and *fac*-$[TeCl_3Br_3]^{2-}$ Raman spectra. All spectra are normalized to their highest intensity.

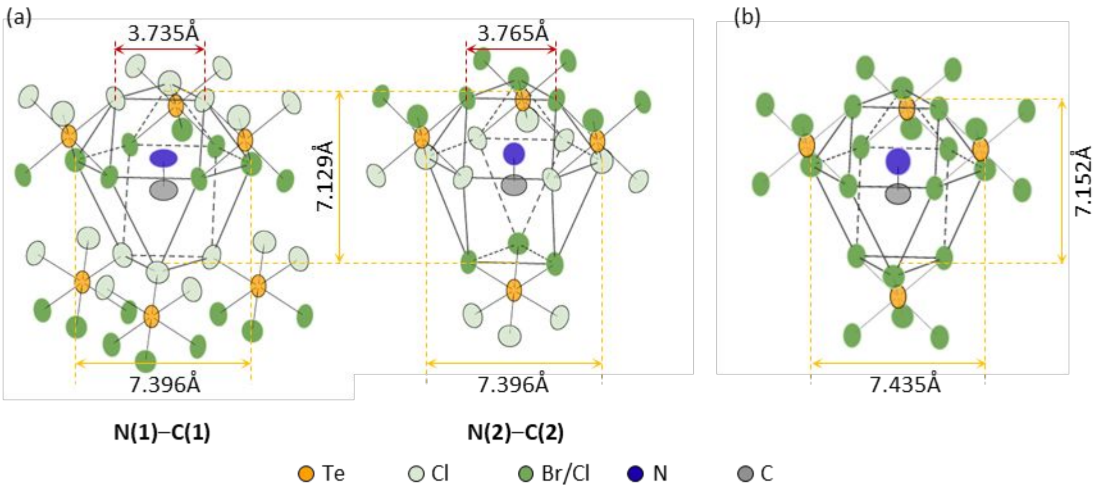

Figure S10 Schematic diagram of the cation cavities in (a) low–Br and (b) high–Br $MA_2Te(Cl_{1-x}Br_x)_6$. The cavity dimensions are labelled. For clarity, hydrogen atoms are not shown.
